# *In vivo* brain imaging with multimodal optical coherence microscopy in a mouse model of thromboembolic photochemical stroke

**DOI:** 10.1117/1.NPh.7.1.015002

**Published:** 2020-01-22

**Authors:** Hubert Dolezyczek, Szymon Tamborski, Piotr Majka, Danuta Sampson, Maciej Wojtkowski, Grzegorz Wilczyński, Maciej Szkulmowski, Monika Malinowska

**Affiliations:** a Nencki Institute of Experimental Biology, Polish Academy of Sciences, Warsaw, Poland; b Nicolaus Copernicus University, Institute of Physics, Faculty of Physics, Astronomy and Informatics, Torun, Poland; c University of Surrey, Surrey Biophotonics, Centre for Vision, Speech and Signal Processing, School of Biosciences and Medicine, Guildford, United Kingdom; d Institute of Physical Chemistry of the Polish Academy of Sciences, Warsaw, Poland

**Keywords:** optical coherence microscopy, photochemical thromboembolic stroke, angiography, cranial window, *in vivo* mouse brain imaging, cerebral blood flow

## Abstract

We used a new multimodal imaging system that combines optical coherence microscopy and brightfield microscopy. Using this *in vivo* brain monitoring approach and cranial window implantation, we three-dimensionally visualized the vascular network during thrombosis, with high temporal (18 s) and spatial (axial, 2.5  μm; lateral, 2.2  μm) resolution. We used a modified mouse model of photochemical thromboembolic stroke in order to more accurately parallel human stroke. Specifically, we applied green laser illumination to focally occlude a branch of the middle cerebral artery. Despite the recanalization of the superficial arteries at 24 h after stroke, no blood flow was detected in the small vessels within deeper regions. Moreover, after 24 h of stroke progression, scattering signal enhancement was observed within the stroke region. We also evaluated the infarct extent and shape histologically. In summary, we present a novel approach for real-time mouse brain monitoring and ischemic variability analysis. This multimodal imaging method permits the analysis of thrombosis progression and reperfusion. Additionally and importantly, the system could be used to study the effect of poststroke drug treatments on blood flow in small arteries and capillaries of the brain.

## 1 Introduction

Global statistics indicate that stroke is the leading cause of human mortality, resulting in nearly 6 million deaths annually worldwide.^1^^,^^2^ Stroke also has a greater impact on disability than any other chronic disease. Moreover, the incidence of stroke is rapidly increasing and is predicted to double by 2030.^3^ There are two major types of stroke, ischemic and hemorrhagic, with the former being almost three times more common than the latter.^4^^,^^5^ Ischemic stroke is caused by an artery occlusion due to a thrombus originating *in situ* or downstream. Thrombolysis is the only treatment approved for ischemic stroke and requires alteplase (rt-PA) administration within a short-time window after clot formation (up to 4.5 h).^6^ However, in 12% of patients treated with rt-PA, early (30 day) readmissions have been observed.^7^

Stroke will continue to be one of the most pressing medical problems and requires new treatment solutions.^8^ To better understand this pathology and test novel therapies, researchers need sophisticated approaches to mimic stroke in animal models. Although many well-established animal (mouse, rat) stroke models have been used for decades,^9^ these models are based solely on postmortem brain histology, making neurovascular monitoring during stroke impossible.

The cranial window implantation procedure has opened up a new area of brain research.^10^ Recently, *in vivo* small animal brain imaging techniques have been developed to provide valuable data, with the potential for translation into human therapies.^11^ The methods currently being used for vascular system visualization include positron emission tomography, magnetic resonance angiography (MRA), transcranial Doppler,^12^ photoacoustic probing,^13^ two-photon microscopy,^10^ and optical coherence microscopy (OCM).^14^^,^^15^

Of these methods, OCM stands out because it provides (i) three-dimensional (3-D) imaging of deep brain tissue at a micron-scale resolution, (ii) a reasonable field of view of several millimeters, and (iii) feasible temporal resolution, enabling real-time measurements.^16^ Furthermore, available angiographic OCM measurement protocols provide blood vessel contrast by utilizing light signal variation that is caused by scattering (due to red blood cell movement).^17^ Thus with OCM, no additional contrast is needed. Angiographic imaging modalities have the potential to deliver high-resolution 3-D maps of brain vasculature at the microcapillary level, as well as record hemodynamic, vascular, and oxygen saturation changes in stroke.^18^^,^^19^ This data can then be systematically analyzed. Moreover, OCM permits real-time and simultaneous *in vivo* visualization of mouse brain structure and vasculature.^20^

An animal model system for comprehensively modeling stroke, as well as monitoring therapy effectivity, should visualize brain structure and vasculature (i) noninvasively, (ii) continuously, (iii) in three dimensions, (iv) at a high resolution, and (v) permit real-time stroke induction during imaging. Previously, we developed a multimodal system combining extended-focus OCM and brightfield microscopy^20^ that was useful for noninvasive measuring microvascular angiography and hemodynamics. We further developed and modified that system using a stroke photoinduction channel in the mouse brain to induce targeted photothrombosis and perform detailed characterization of focal ischemia. This multimodal approach allows for 3-D, noninvasive, and high-resolution structural and angiographic brain monitoring during a stroke photoinduction procedure with a relatively high temporal resolution of <20  s per 3-D data set. With an OCM microscope, Bessel beams measure an extended depth of field compared to a classical Gaussian beam.^21^ In our configuration, the axial resolution is 2.5  μm within 800  μm of the axial range. Thus in contrast to a standard scanning confocal microscope, the Bessel beam configuration allows for the detailed visualization of mouse dura, arachnoid, and pia mater structure, as well as cortical layers.^20^ This configuration also permits angiographic imaging of subarachnoid space blood vessels without requiring mechanical axial scanning.

We present in this paper an induced mouse model of thromboembolic photochemical stroke,^22^ achieved by injecting animals with a photoactive dye [Bengal Rose (BR)] and precisely localizing photoinduction within a specific brain artery. Analysis of the resulting OCM data revealed insights into ischemia development over time. We additionally used morphological techniques to delimit and to analyze stroke-associated degenerative changes in the mouse brain. For postmortem ischemic brain evaluation, we used common, well-established techniques [i.e., hematoxylin and eosin (HE), Fluoro-Jade B (FJ), and triphenyltetrazolium chloride (TTC)]. Postmortem evaluation combined with the OCM data provided comprehensive information about mouse brain conditions before and after photochemical stroke induction.

## 2 Materials and Methods

### 2.1 Experimental Setup

**Fig. 1:**
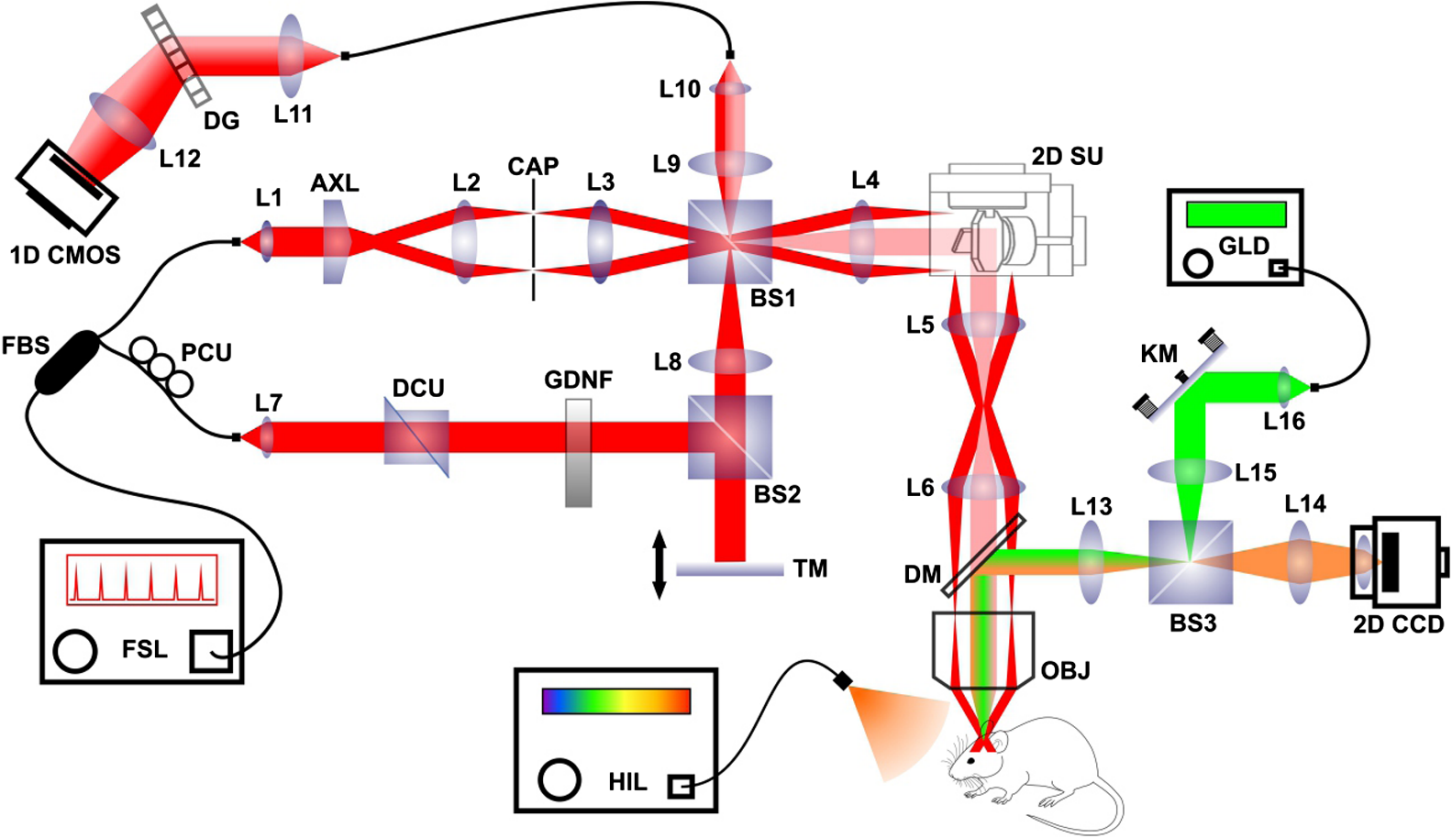
Multimodal OCM system scheme. FSL, femtosecond TI:Sa laser; HIL, halogen illuminator; GLD, green laser diode; 1-D CMOS, line-scan CMOS camera; 2-D CCD, array CCD camera; L1–L16, lenses; AXL, axicon lens; OBJ, objective lens; GDNF, gradient density neutral filter; PCU, polarization control unit; DCU, dispersion control unit; TM, translation mirror; KM, kinematic mirror; FBS, fiber beamsplitter; DG, diffraction grating; CAP, circular aperture; DM, dichroic mirror; BS1–BS3, glass cube beamsplitters; 2D SU, 2-D galvanometric scanning unit. Beam path color coding: Bessel beam illumination (red), Gaussian beam detection (light red), brightfield detection (amber), and focal stroke photoinduction illumination (green).

The multimodal imaging system used in this study is presented in Fig. 1. Extended-focus OCM scanning permits 3-D structural and angiographic mouse brain imaging. The brightfield microscope channel permitted precise animal positioning and real-time superficial vasculature imaging. These modalities were previously described by Tamborski et al.^20^ Briefly, a femtosecond laser (Fusion Femtolasers, Austria) centered at 795 nm and with a 130-nm full bandwidth was used for OCM scanning. The interferometer was constructed using the Mach–Zehnder scheme. In the illumination path, we used an axicon lens (apex angle: 176 deg, Thorlabs, Newton, New Jersey, USA, Part No. AX252-B) to produce the Bessel beam, which was delivered by two telescopes to an Olympus BX61VS microscopy system [objective lens, 10×; NA, 0.3, Olympus (Japan)]. To obtain a proper Bessel beam profile, a circular beam blocker was placed in the focal plane of the first telescope to effectively eliminate residual light from axicon tip imperfections. Lateral laser beam scanning was accomplished using a pair of galvo-scanners (Cambridge Technology, Bedford, Massachusetts, USA). The microscope was set to epi-mode with standard Gaussian detection. Backscattered light was recombined with reference arm-propagated light and coupled to the spectrometer (diffraction grating, 1200 lpmm; Wasatch Photonics, Morrisville, North Carolina, USA; camera, 70 kHz and 2048 pixels; Basler Sprint, Exton, Pennsylvania, USA). The setup characteristics are presented in Table 1.

**Table 1:** Key OCM parameters.

Parameter	Value
Axial resolution	2.5 μm
Extended-focus-range lateral resolution	2.2 μm (1.75 μm minimum at the focal center)
Extended focus range	800 μm
Object light power	11 mW
Maximum acquisition rate	70 kHz
Number of OCM spectrum pixels	2048
Sensitivity	92 dB

The brightfield microscope was combined with the OCM system using a dichroic mirror. The lens system, combined with a commercial CCD camera lens with manual focus (Logitech, Switzerland), permitted real-time HD video recording (30 frames per second) for precise animal positioning relative to the scanning beam and two-dimensional (2-D) stroke progression monitoring. The third part of the system, which was used for local photoinduction, shared a part of the optical path with the brightfield microscope and was connected to the OCM microscope using the same dichroic mirror. A laser diode (Thorlabs), centered at 520-nm and with 10-mW of power, was used for BR (Sigma Aldrich, St. Louis, Missouri, USA) photoactivation. For precise blood vessel targeting, the beam waist in focus was adjusted using the system collimator. A kinematic mirror was used to localize the beam within the entire OCM field of view.

### 2.2 Scanning Protocol and Data Processing

The measurement protocol was designed for optimized angiographic analysis. The lateral scanning area was 1.1 mm (fast scanner direction, X) × 1.1 mm (slow scanner direction, Y). The Y range was sampled with 300 points and in each Y scanner position the set of six overlapping B-scans consisting of 300 A-scans was acquired. Thus a four-dimensional (X,Y,λ,t) data set was the result of every measurement, which allowed for further pointwise 3-D (X,Y,Z) differential complex signal analysis of angiographic imaging data as described previously. The A-scan repetition time was 20  μs. The total sample scanning time was 9.7 s, whereas the total measurement time (including time of acquisition and dead time required for data transfer to hard drive) was 18 s which determined the time resolution of the 4-D imaging. The scanning protocol parameters are presented in Table 2.

**Table 2:** Scanning protocol parameters.

Parameter	Value
Number of B-scans	300
Number of A-scans per B-scan	300
B-scans oversampling	6
X range	1.1 mm
Y range	1.1 mm
Z range	2.12 mm (in air)
Acquisition time per A-scan	18 μs
Repetition time per A-scan	20 μs (acquisition+dead time)
3-D angiographic imaging temporal resolution	18 s

The acquisition was carried out using custom-written software developed in a C++ environment. It provided full control of the experiment and implementation of the designed scanning protocols by synchronizing the spectrometer line scan camera and the galvanometric scanners. Data analysis was performed using nonstandard algorithms created in the LabView environment. The software enabled structural 3-D reconstruction of scanned regions, as well as 3-D angiographic maps. The initial processing of raw OCM data included standard steps: spectrum linearization in k space, residual dispersion correction, and Fourier transform (fast Fourier transform) of each recorded spectrum. Thus obtained A scans of complex value have been cropped to the area of interest in the Z axis, i.e., signal rejection from the glass and optionally corrected for potential tilt. Structural 3-D images were obtained by further averaging the complex value A-scans in the oversampling set (six A-scans). Angiographic analysis was carried out according to the procedure described previously.^23^ First, the phase of the complex A-scans was corrected in the oversampled set for potential bulk motion based on the phase variation obtained from the vessel-free area of the B-scan of interest. Second, the complex differences were calculated for each pair of the A-scans, and the absolute value of the averaged difference was produced. Motion (angiographic) B-scans were produced by an angiographic differential algorithm obtained from the six scans repeated for each B-scan position. Thus the intensity of the obtained angiographic B-scans coded the change in phase due to the blood flow. Motion B-scans formed the 3-D angiographic set of data. For the purposes of presenting data in the manuscript, cerebral cortex depth was coded in a false color to allow visualization of the development of ischemia in both the superficial and deep cortical layers.

To quantify blood flow, we measured changes in the total Angio-OCM signal present in an arbitrarily selected volume (the same at each time point) of imaged tissue. Integration of the total Angio-OCM signal at one time allowed for comparison of temporal flow dynamics. We calculated a dynamic change in total flow at three cortical depths, selected due to functional differentiation of cerebral vessels. The depth range of 0 to 160  μm mainly refers to meningeal blood vessels, the range 160 to 320  μm to penetrating arterioles, and the range below 320  μm refers mainly to capillaries. After segmenting these layers with Angio-OCM, the threshold level was adjusted to reject the background noise by selecting those phasor differences that corresponded to the blood flow in the selected vessels and capillaries. Then Angio-OCM intensity values were integrated. This procedure was repeated for all volumes obtained in the time sequence. To avoid the influence of strong signals from large superficial vessels, we removed the appropriate locations by masking the Angio-OCM signals with an inverted image of the large vessel. For the purpose of quantitative comparison, the angiographic maps obtained from the measurements, performed 24 h after stroke induction, were scaled by equalizing the noise levels and compensating for the depth-dependent Angio-OCM signal intensity differences based on the structural 3-D images. Values of the flow after 24 h were generated by averaging OCM data from the total 5 min of scanning.

### 2.3 Animals

All procedures were performed according to the rules established by the First Local Ethical Committee on Animal Research in Warsaw. These rules are based on national laws that are in full accordance with the European Union directive on animal experimentation.

### 2.4 Cranial Window Implantation

Twenty certified male C57BL/J6 mice (age, 6 to 10 weeks old; weight, 20 to 25 g) were used in the experiments. Two mice were excluded due to technical failure. Photochemical stroke was induced in one group of mice (n=14). Of these mice, 10 underwent FJ staining and four underwent TTC staining. The second group of mice (n=4) served as a control. The mice were prepared for imaging by implanting a cranial window over the parieto-temporal lobe, centered on the barrel cortex of the right hemisphere. The cranial window was centered at 2.5 mm posterior to bregma. The mice were placed in a stereotaxic frame and deeply anaesthetized with isoflurane (4% for induction; 1.5% to 2% for surgery). Dexamethasone (0.2  mg/kg) and carprofen (5  mg/kg) were administered subcutaneously to prevent an inflammatory response and brain edema. A warming pad with an anal probe containing an automated temperature control feedback system was used for monitoring body temperature, which was maintained at ∼37°C during surgery and ischemia. After removing the skin over the top of the skull, a drop of 1% lidocaine:epinephrine (1:100000) solution was applied onto the periosteum to avoid excessive bleeding or pain. A circular portion of the skull (diameter, 4 mm) was removed to expose the dura mater. The dura mater was covered with 0.9% NaCl and a cover glass (diameter, 5 mm) was attached with cyanoacrylate-based glue and sealed with dental acrylic. After the surgical procedure, the mice were transported to cages and monitored until total recovery.

### 2.5 Stroke Induction and *In Vivo* OCM Monitoring

**Fig. 2:**
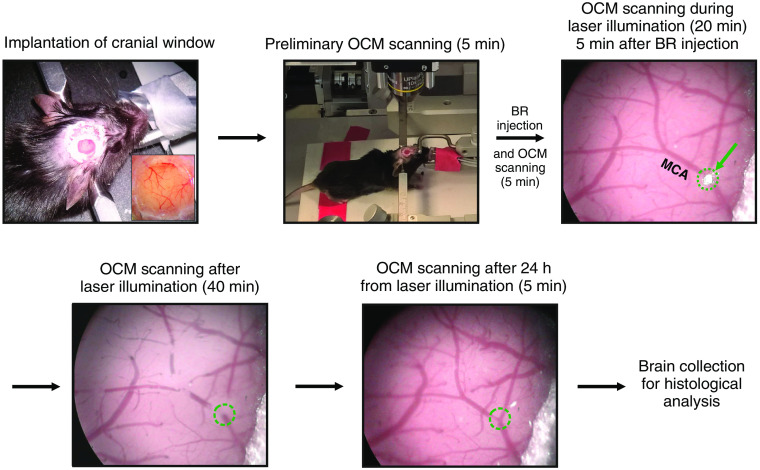
Experimental procedure block diagram. Green arrow shows the laser beam focus (diameter∼120  μm) positioned at the MCA bifurcation. The focus is surrounded by a dotted green line.

After cranial window installation, OCM measurements were performed in 14 mice at ≥1 week of recovery. Each animal was placed in a stereotaxic frame with a warming pad and was deeply anaesthetized with isoflurane (4% for induction; 1.5% to 2% for the procedure). The stereotaxic frame was then positioned in the OCM setup using a custom-made three-axis platform. The animal’s brain window was accessed by the extended focus of the imaging system. The long working distance (7.5 mm) of the 10× objective lens allowed for precise and comfortable animal positioning. To induce photoactive stroke, initially BR, 100  mg/kg body weight in saline (25  mg/mL), was administered intraperitoneally. Then after 5 min, when the BR dye reached the blood circulation,^24^ photoactivation was initiated using a 520-nm laser diode beam focused on the bifurcation of the middle branch of the middle cerebral artery (MCA), located ∼1 to 2 mm posterior to bregma. The beam diameter was adjusted to the diameter of the artery. Artery illumination continued for 20 min. Clot formation kinetics were monitored with the brightfield microscope. OCM measurements were acquired continuously before BR injection (5 min), after BR injection (5 min), during green laser illumination (20 min), and after laser illumination (40 min), and were repeated 24 h later (5 min of continuous recording). Structural B-scan transformation produced the following images: phase-variance (motion) B-scans and structural and angiographic *en face* projections of the imaged region. The experimental procedure is summarized in Fig. 2. To histologically verify the photoinduction model, some animals in the control group received only BR without laser illumination (+BR-L), whereas other animals only received laser illumination without BR (−BR+L).

### 2.6 Histology

Histological staining was performed 24 h after stroke induction. The mice were anesthetized with pentobarbital and perfused with warm (37°C) phosphate-buffered saline (PBS) for 5 min followed by 4% paraformaldehyde in PBS for 15 min (at room temperature). The brains were postfixed overnight in the same fixative solution.

### 2.7 HE Staining

Seven brains were embedded in paraffin and cut on a microtome into serial coronal sections at a thickness of 4  μm. The paraffin sections were mounted on SuperFrost (Fisher Scientific, Hampton, New Hampshire, USA) slides, air dried on a slide warmer at 58°C for at least 30 min, and then deparaffinized with xylene. Following this, the sections were hydrated by passage through water baths with decreasing alcohol concentrations (2 min in each bath of 100%, 80%, or 50% alcohol and water), stained in Mayer’s hematoxylin (Diapath, Martinengo, Italy) for 6 min, and washed in running tap water for 5 min. Next, the slides were stained in Shandon Eosin Y (Thermo Fischer Scientific, Waltham, Massachusetts, USA) for 2 min, washed in tap water for 2 min, dehydrated in baths with increasing alcohol concentrations (1 min in each bath of 50%, 80%, and 100% alcohol), and cleared in xylene. Finally, the sections were mounted in Shandon Consul-Mount media (Thermo Fischer Scientific), which is a nonaqueous, nonfluorescent plastic mounting media.

### 2.8 FJ Staining

For FJ staining, we used brains from 14 mice. Seven brains were transferred to 30% sucrose in 0.01 PBS and kept in a refrigerator for 3 to 5 days. Serial sections were cut at 40-μm intervals in the coronal plane using a freezing microtome. Additionally, we stained paraffin sections from seven mice used for HE staining. For 2 min, paraffin or frozen section slides were immersed in a solution containing 1% sodium hydroxide in 80% alcohol (20 mL of 5% NaOH added to 80 mL absolute alcohol). Following this, the sections were immersed in 70% alcohol for 2 min, 50% alcohol for 2 min, and distilled water for 2 min. Next, the slides were transferred to a 0.06% potassium permanganate solution for 10 min, rinsed in distilled water for 2 min, and stained with FJ (final concentration, 0.0004% in a 0.1% acetic acid vehicle). After 20 min in the staining solution, the slides were rinsed for 1 min across three distilled water washes. Excess water was removed by paper towel. The slides were then placed on a slide warmer set to ∼50°C until fully dry (5 to 10 min). The dry slides were cleared by immersion in xylene for at least 1 min before coverslipping with Shandon Consul-Mount.

### 2.9 TTC Staining

Four mice were euthanized and their brains were quickly isolated, placed in a brain matrix (Zivic Instruments, Pittsburgh, Pennsylvania), and serially sectioned into 1-mm-thick coronal slices. Brain isolation and slicing were completed within 10 min of decapitation. The brain slices were stored in PBS for <3  min at room temperature until they were transferred to a TTC incubation medium (Sigma Aldrich). A solution of 0.1% TTC dissolved in PBS was immediately used for brain slice incubation at 37°C for 30 min. After staining, the slices were washed in PBS three times (1 min per wash) and fixed in 4% paraformaldehyde for 30 min. A digital camera was used to acquire images of the stained brain sections. The untreated left hemispheres served as controls.

### 2.10 3-D Infarct Reconstruction

To examine the shape and volume of the infarct region, a series of 40-μm FJ-stained sections from one stroke-induced mouse brain (M10) were three-dimensionally reconstructed. Twenty-nine serial sections containing the infarct, which was identified by the FJ green fluorescence of degenerating neurons, were imaged with a fluorescent microscope (Olympus BX61VS with a TRITC filter and 10× objective lens; VS-ASV software, Olympus, Japan). The images were converted to 8-bit grayscale, inverted to negative and subjected to brightness and contrast adjustments to enhance infarct visibility. Subsequently, the images underwent 3-D reconstruction. After down-sampling to a 20  μm per pixel resolution and smoothing with a 2×2  pixel median filter, the images were processed according to the “Graph-Based Sequential Alignment” workflow,^25^ which allows for accurate 3-D reconstruction that mitigates section distortions.^26^ Briefly, the images were sequentially aligned, starting from the middle of the stack toward either end using rigid transformations and cross-correlation as a similarity metric. Subsequently, the assembled image volume was manually aligned to the C57B1/J6 Waxholm Space of a mouse brain atlas,^27^ which is based on high-resolution magnetic resonance imaging (MRI). The alignment was performed using the “Image registration” feature of ITK-SNAP software.^28^ Finally, the 3-D-reconstructed infarct region was manually outlined using orthogonal views of the reconstruction in ITK-SNAP.

## 3 Results

### 3.1 Structural and Angiographic OCM Imaging

**Fig. 3:**
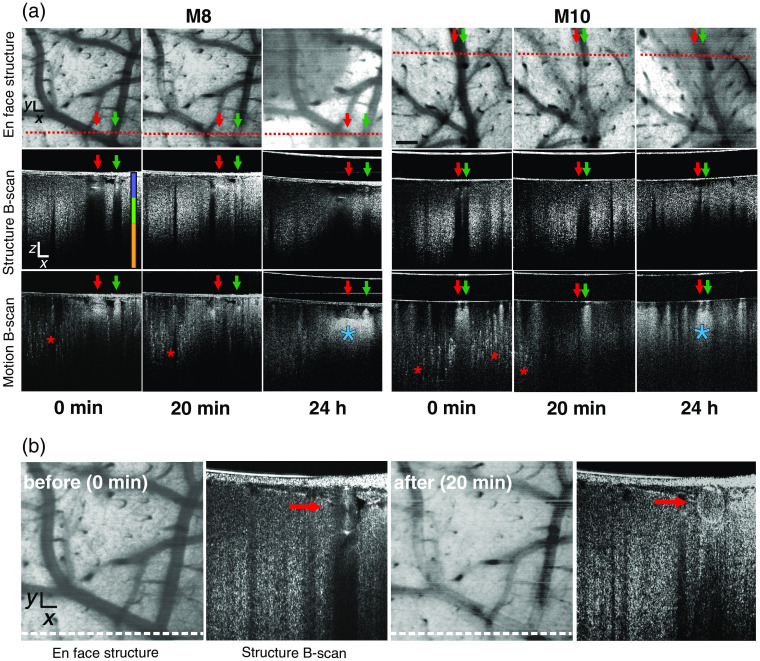
OCM images of brain regions pre- and poststroke induction in M8 and M10. (a) Blood flow changes are visualized at three time points: 0 min (before stroke), 20-min poststroke, and 24-h poststroke. The red dotted line on the *en face* images indicates the location of the structural and motion B-scans. Red and green arrows indicate large vessels. The red asterisk indicates small vessels, and blue asterisk indicates enhanced signal related to reperfusion. The vertical three-color line shows three cortical depths (violet 0 to 160  μm, green 160 to 320  μm, orange below 320  μm), in which blood flow was calculated and presented in Fig. 4(b). (b) *En face* structure and structure B-scans showing a difference in the scattered signal pattern in the MCA before and after occlusion (dotted white lines indicate cross-sections corresponding to the B-scans; red arrows indicate the lumen of the MCA branch). Scale bar=100  μm.

To monitor thromboembolic photoinduced stroke progression, structural and angiographic OCM imaging was combined with continuous brain surface viewing, using a brightfield microscope. OCM scanning produced coronal B-scans of the brain. Stacks of B-scans were flattened in the z axis, which was represented by the mean intensity value of each A-scan. These stacks produced 2-D structural *en face* projections of the imaged region. Structural B-scans provided information on dynamic structural changes, particularly in blood vessel cross sections. The brightness of the structural B-scans represented the intensity of light scattered back from the object. Before stroke photoinduction, cross sections of vessels with normal blood flow were characterized as having a strong signal with an underlying dark shadow, i.e., lack of signal (see Fig. 3, the red and green arrows on 0-min structure B-scans from representative mouse images M8 and M10). The shadow is caused by light scattering due to red blood cells. This scatter is of a much greater magnitude than the scatter in the adjacent tissue. These vessels and their course are visible on *en face* projection as homogenously dark structures. In motion B-scans, blood flow appears as a strong bright signal (see the red and green arrows on 0-min motion B-scans for M8 and M10 in Fig. 3) with bright shadows beneath the vessels. Also in motion B-scans, a uniquely dotted bright signal can be used to differentiate small vessels and capillaries (see the red stars on 0-min motion B-scans for M8 and M10 in Fig. 3). Analysis of cortical blood flow dynamics during photoinduced stroke revealed changes in sequential clot formation stages. At 20 min from the start of laser illumination we observed no blood flow in M8 superficial arteries on B-scan images. However, in M10, blood flow in one vessel (vein; see green arrow in Fig. 3) was normal, whereas no blood flow was observed in the second vessel (artery; see red arrow in Fig. 3). Similar findings were also observed in the corresponding M10 motion B-scans, where only the vein had a strong signal (see green arrow in Fig. 3). The structural B-scan revealed the presence of a clot in the lumen of the MCA, visible as a homogenous scattered signal with a slight shadow below; this pattern is clearly distinguishable from the appearance of the lumen before stroke [Fig. 3(b)]. After 24 h, a partial reperfusion of the MCA branches of both mice was visible on structural *en face* projections and motion B-scans (see the red and green arrows on 24-h scans for M8 and M10 in Fig. 3). Interestingly, in motion B-scans, there was no signal, which reflects that small vessel flow was not restored. Additionally, in motion B-scans, below the cross sections of both vessels, we noticed significant changes in the shape and brightness of the shadows [see blue stars on 24-h motion B-scans for M8 and M10 in Fig. 3(a)] compared with their appearance in both normal tissue and tissue 20 min after stroke induction. This can be caused by structural changes in the cortex due to temporary hypoxia.

**Fig. 4:**
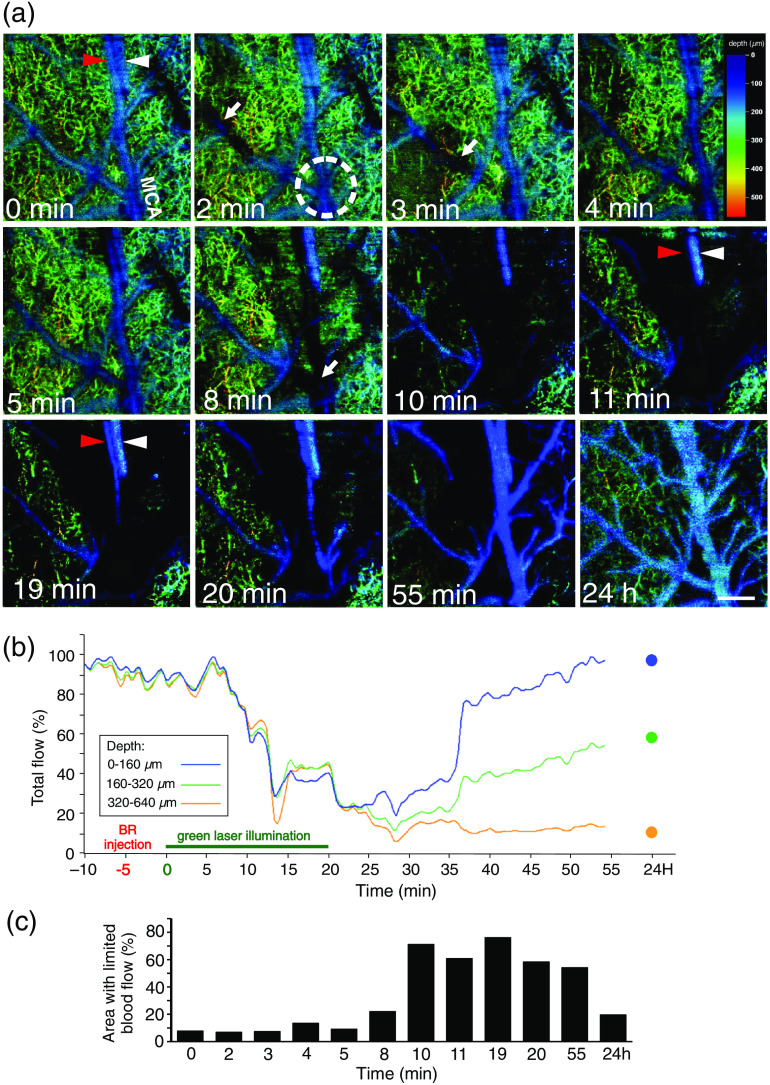
Angiographic OCM maps illustrating the vascular response and relative changes of blood flow within 24 h after stroke. (a) The white dotted circle indicates the MCA bifurcation where the laser beam was focused. White arrows show the occluded portions of MCA branches with limited blood flow. Arrowheads show two parallel vessels, the MCA branch (red arrowhead) and vein (white arrowhead), at three selected time points: 0, 11, and 19 min. Blood flow is normal in the vein and altered in the MCA branch. The color scale bar indicates the depth from the brain surface. Scale bar=200  μm. (b) Changes in the total intensity of the angiographic signal in three acquired volumes at different depths of the cerebral cortex. (c) The area with limited blood flow (black spots) at the time points shown in [Fig. 4(a)] is represented as a percentage of the positive-signal area, estimated using ImageJ.

Stacks of motion B-scans flattened in the z axis provided 3-D angiographic *en face* projections of the scanned ischemic region. These angiographic projections showed the dynamics of blood flow caused by thrombi formation and migration during photoinduced stroke. As seen in Fig. 3(b), intensity variations in the structural and motion B-scans reflect impeded movement of red blood cells within pial microvessels, indicating thrombus formation. In Fig. 4(a), we present *en face* angiographic maps at selected time points with color coding of vessel depth. In the second minute of laser illumination, areas with limited blood flow (black spots) occurred. The localization of these areas changes, which can be seen in the example of the MCA branch, from its distal region (2 min) to a more proximal position at 3 min [white arrows in Fig. 4(a)]. Occluded MCA branches limited the blood flow within the related vascular network. During the tenth minute of lighting, there was a sharp decrease in blood flow, and the area with restricted flow increased to about 60% [Fig. 4(c)]. In most of the scanned region, there was no arterial or capillary blood flow, with the exception of a vein running along the occluded artery [see red and white arrowheads on the 11-and 19-min scans in Fig. 4(a)]. At ∼1  h after stroke induction, partial recanalization of the main MCA branches occurred; however, occluded small vessels and capillaries remained. At 24 h after stroke, we noticed further blood flow improvement in the main arteries and in several deeper small arteries. Figure 4(b) and Video S1 show changes in the total intensity of the angiographic signal in three segments of the acquired 3-D volumes located at different depths (0 to 160, 160 to 320  μm, and 320 to 640  μm). The plots show the temporal variability of the total flow, indicating the hemodynamics of the three stages: normal (with total flow values maintained at the same level), stroke (total flow values dropped to about 20% of the original value over 5 min and remained low for the next 20 min), and recovery (after flushing the clots, the Angio-OCM signal in large vessels returned to its initial value over 20 min, while the total flow present in the small capillaries remained much lower). Interestingly, in our measurements, the flow in the large vessels was almost completely restored, but flow in the middle segment was restored in only ∼50% of vessels. It is also evident that the stroke effect was strongest in the deepest segment, containing cortical microvessels.

**Fig. 5:**
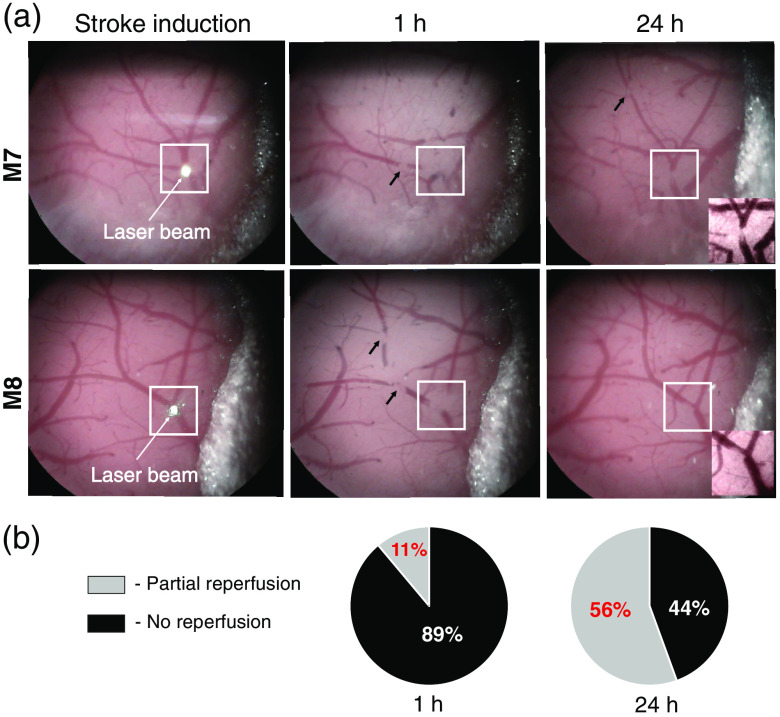
Reperfusion of MCA branches within the stroke induction region. (a) Brightfield microscope images showing the brain surface in two representative mice (M7 and M8) during stroke photoinduction and at 1 and 24 h postinduction. White squares indicate the regions, in which the laser beam was focused on the MCA bifurcation. Black arrows show clot positions in the vessel lumen. Insets show magnifications of the occluded MCA without reperfusion (M7) and with partial reperfusion (M8) at 24 h after stroke photoinduction. (b) Percentage of mice with partial or no reperfusion (n=9).

We also monitored recanalization progression in the main branches of the MCA at stroke induction and at 1 and 24 h after stroke. We used the brightfield microscope because it clearly showed decreases in the superficial vessel diameter. In the MCA bifurcation, spontaneous partial reperfusion was observed in 11% and 56% of the animals at 1 and 24 h after stroke induction, respectively [Figs. 5(a) and 5(b)]. No reperfusion was observed in 89% and 44% of the animals (n=9), respectively [Fig. 5(b)].

Brightfield microscopy video recording, at the beginning of the laser illumination, showed that small thrombi tended to detach from the vessel walls and move peripherally in the bloodstream within the MCA vascular network. Moreover, bidirectional clot migration occurred during laser illumination for 20 min and after 1 h (see Video S2). After 24 h, the number of clots visibly diminished.

### 3.2 Histological Verification of the Infarct Region

**Fig. 6:**
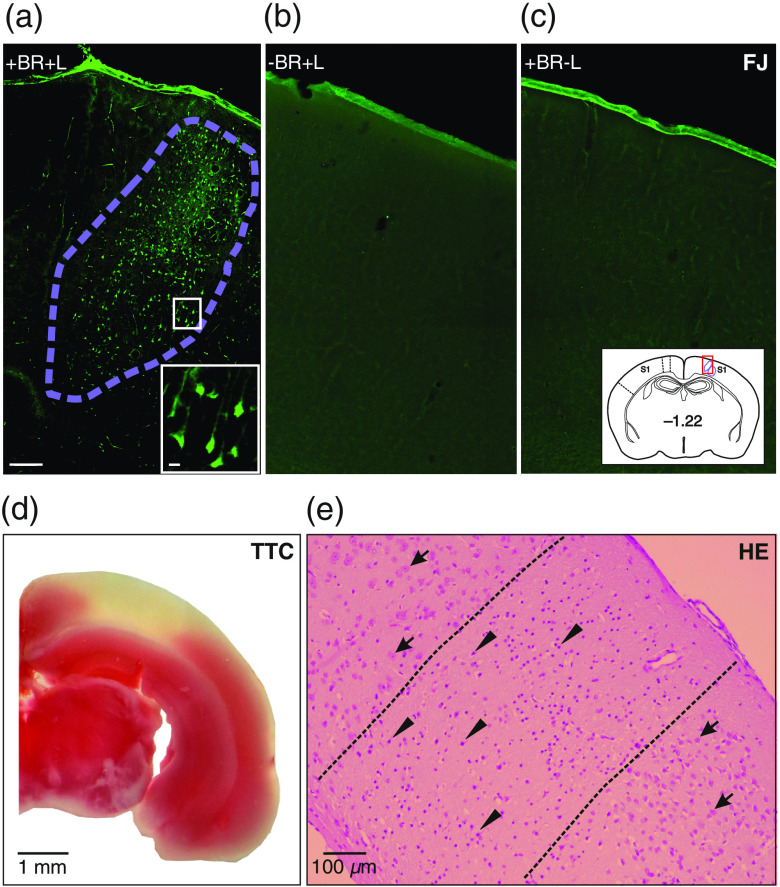
Histological infarct region verification by FJ, HE, and 2,3,5-triphenyltetrazolium chloride staining (TTC). (a) Photoinduced stroke core (outlined by violet dotted line), with inset showing degenerating pyramidal neurons. (b)–(c) FJ staining in control without Bengal Rose (−BR+L) or laser illumination (+BR-L), respectively. Photographs (a)–(c) are from the same region (inset, red square). (d) TTC staining, with infarct visible in white. (e) HE infarct staining (outlined by black dotted line), with arrowheads indicating degenerating neurons and arrows indicating normal neurons.

To determine infarct extent, sections were stained with FJ to visualize degenerating neurons [Fig. 6(a)]. In control animals, we found no FJ-positive neurons [Figs. 6(b) and 6(c)]. To verify the effectiveness of the applied photoinduced stroke model, we performed additional HE and TTC staining. TTC staining revealed a white infarct region with unreduced TTC surrounded by viable tissue that stained red due to reduction of TTC to formazan [Fig. 6(d)]. HE staining showed a core infarct region characterized by the presence of shrunken, dark red degenerated neurons surrounded by normal brain tissue with normal pyramidal neurons [Fig. 6(e)].

### 3.3 Infarct Distribution

**Fig. 7:**
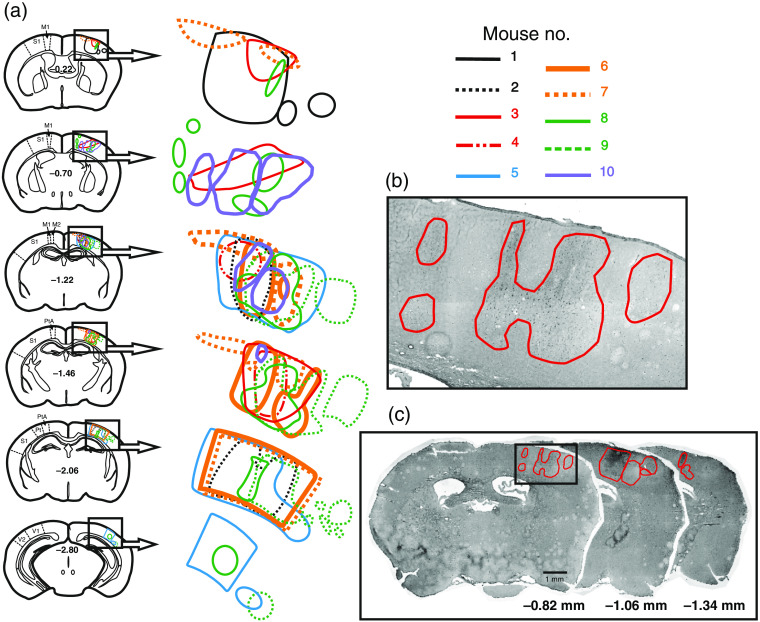
Infarct reconstruction. (a) Schema illustrating cerebral infarct shapes and medio-lateral distribution on representative coronal sections at six anterior–posterior bregma levels. At the anterior level, all infarcts were primarily situated in the somatosensory cortex, whereas at more posterior levels, infarcts were localized in the parietal association and visual cortices. M1, primary motor cortex; M2, secondary motor cortex; S1, primary somatosensory cortex; PtA, parietal association cortex; V1, primary visual cortex; V2, secondary visual cortex. (b) Magnified infarct regions from section −0.82  mm. (c) Representative sections from anterior, middle, and posterior levels. Infarct regions are surrounded by red lines.

The infarct regions of 10 mice were delineated from FJ-stained sections. Infarcts occupied the dorsal-lateral convexity of the parieto-temporal cortex between −0.22 and −2.8  mm from bregma [Fig. 7(a)]. At the anterior level, all infarcts were mainly situated in the somatosensory cortex. At more posterior levels, the infarcts were located in the parietal association and visual cortices. Although the laser illumination target was limited to the diameter of a single MCA branch, infarct distributions and shapes were heterogeneous. Some infarcts extended through all cortical layers, whereas others were situated in either superficial or deep cortical layers [Figs. 7(b) and 7(c)]. Most infarcts had irregular shapes. In Fig. S1 in the Supplementary Material, a diagram depicts the anterior–posterior positions of FJ-stained infarcts in individual mice.

### 3.4 3-D Infarct Reconstruction

**Fig. 8:**
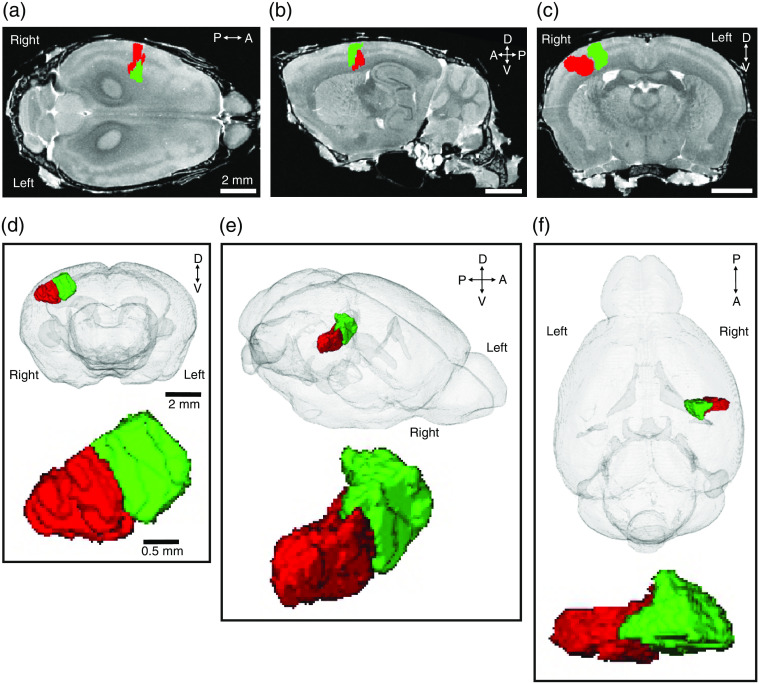
3-D infarct reconstruction in a representative mouse (M10) registered to the high-resolution-MRI mouse brain. Examples of infarct locations on (a) horizontal, (b) sagittal, and (c) coronal images. Infarct shape is shown from (d) anterior, (e) dorso-lateral, and (f) dorsal positions. Green and red colors indicate two partially separated parts of the infarct.

We measured the volume ($1.4\;{\mathrm{mm}}^{3}$, which is 1.1% of a hemisphere) as well as maximum anterior–posterior length (1.1 mm), lateral–medial width (2.4 mm), and height (1.4 mm) of a 3-D infarct reconstruction. The infarct territory was subdivided into two main parts, partially separated from each other by a narrow groove (see red and green structures in Fig. 8). To determine the infarct location in relation to other brain structures, we registered the infarct to a 3-D C57Bl/J6 mouse brain atlas reconstructed from high-resolution MR images. The location of the infarct within the brain can be viewed in the horizontal [Fig. 8(a)], sagittal [Fig. 8(b)], and coronal [Fig. 8(c)] planes. To more precisely show the 3-D infarct within the brain, we created a video of the coronal sections (see Video S3).

## 4 Discussion

We used a thromboembolic photochemical stroke model to monitor blood flow in ischemic cortex. To induce thrombi formation, BR photoactive dye was intraperitoneally injected in order to reach the bloodstream. This was followed by green laser MCA bifurcation illumination. The incorporation of an optical green laser system into the OCM setup allowed for two benefits: beam focusing on a specific vessel and precise beam diameter adjustment. Free oxygen radicals released during laser irradiation indirectly caused aggregation of platelets, which eventually formed a thrombus occluding artery.^29^^–^^32^ For brain imaging, we implanted a cranial window in the mouse skull. This commonly used technique allows poststroke cortical vasculature to be observed for prolonged periods, up to several months. However, as with any surgical intervention, this procedure induces local inflammation, which can last for 3 months.^10^ OCM is an interferometric imaging method that is highly sensitive to scanned object movement, and, as such, can be used for localized motion detection. We applied a carefully designed 3-D scanning protocol that provided sixfold, oversampled B-scans. These scans were then subjected to differential analysis, which resulted in angiographic B-scans that revealed blood movement. However, despite proper mouse head immobilization using a stereotaxic frame, some small movements (e.g., breathing, heart beating) could appear in angiographic images as unwanted artifacts.

Structural B-scans produced by OCM measurements can be used to visualize vessel blood flow. We observed different scattered signal patterns within vessels before stroke, after stroke induction, and during reperfusion after 24 h. Before occlusion, we observed the characteristic “butterfly” pattern on vessel cross sections with a black shadow underlying the vessel; this is thought to be caused by fast laminar flow.^20^ In vessels occluded by a thrombus, the “butterfly” pattern and black shadow were not present, indicating a lack of red blood cell movement within the vessel lumen. During reperfusion with limited blood flow, the scattered signal was weaker. Another scattered signal pattern was observed on motion B-scans. Before stroke, vessels and capillaries had strong signals. However, during spontaneous reperfusion 24 h later, the capillary signal was no longer visible. Interestingly, after 24 h, we observed a strong signal below some vessels during reperfusion, which reflected changes in tissue scattering properties. This finding may be related to vasogenic edema or tissue degeneration. Similar observations were described by Yang et al.^33^ and Choi et al.^34^ Similar to Yang et al.,^33^ we also observed enlarged recanalized artery diameters.

OCM structural *en face* projections provided information about the location, size, and migration of thrombi. Our real-time recording approach allowed for 3-D clot migration monitoring, which was previously impossible due to the lack of appropriate techniques. Spontaneous clot migrations have also been observed in humans.^35^ Using preinterventional computed tomographic angiography and MRA, Kaesmacher et al.^35^ showed that spontaneous clot migration occurs in 30% of patients who underwent endovascular thrombectomy, regardless of rt-PA administration. In our study, angiomaps of 3-D vascular networks were collected in real time (temporal resolution, 18 s) during stroke and reperfusion, which permitted the imaging of vascular network blood flow changes, thrombi formation, and bidirectional migration.

OCM signals should be interpreted with caution because they are exposed to the noise in phase as well as the amplitude. This can be caused by a number of factors, e.g., the momentary change of blood density or the orientation of blood components. The angiographic representation of the blood flow is thus disturbed and its quantification is burdened by the error. To limit this effect, in our approach we repeated the acquisition of every B-scan six times. This provided five differences in the OCT phasors which then were averaged and as the result the error decreased to some extent. The residual variation of the angiographic signal manifested as a random modulation of the plots in Fig. 4(b). Quantitative flow assessment has limited use when the angiographic OCM method is applied. However, if we can guarantee a comparable level of hematocrit and a repeatable orientation of the blood vessels, then this type of analysis may yield useful results.^23^

To measure blood flow during stroke and reperfusion, we used Angio-OCM instead of the OCT Doppler. There are restrictions on the use of the OCT Doppler for *in vivo* tests on biological structures.^36^ The first is that this technique allows for the absolute measurement of only one flow velocity vector element—parallel to the probe beam. The angle between the sampling beam and the vessel of interest is required to calculate the full flow velocity vector. Second, most of the vessels are horizontal. In this orientation, the Doppler angle is very small because of the axial component of the flow velocity. Thus the precision of determining its value is significantly limited by noise. Due to the limited accuracy of the Doppler-OCT method, angiographic imaging of the vascular system using Angio-OCM^37^ seemed more suitable for our purposes. This technique provides detailed maps of blood vessels that can be used for effective segmentation, statistical quantification, and observation of relative flow changes. It usually works at longer time scales than Doppler OCT because it is based on the decorrelation of spots caused by the flow of scatterers, mainly red blood cells, using not only phase information but also light intensity. In contrast to Doppler techniques, the horizontal orientation of the vessels promotes speckle decorrelation, thus providing increased dynamic contrast of vessels on angiographic maps.

The angiographic data were confirmed by surface vessel blood flow observations recorded by the brightfield microscopy. We observed spontaneous partial reperfusion in 11% and 56% of animals at 1 and 24 h after stroke induction, respectively. Similarly, in a study using another mouse thromboembolic stroke model, spontaneous reperfusion was observed within 3 h after stroke in 80% of animals.^38^ In contrast, Yang et al.^33^ observed reperfusion due to spontaneous thrombolysis and recanalization 4 days after photoinduced stroke in rats. These divergent findings may be due to differences in the size of the illumination areas (1-mm diameter versus single artery illumination). In humans, spontaneous reperfusion was observed in 60% of patients 7 days after stroke and in 77% of patients 14 days after stroke.^39^

For ischemic cortical region analysis, we stained postmortem brain slices with HE, TTC, or FJ. FJ staining was more sensitive than TTC staining, which indicates dehydrogenase activity and underestimates infarct size.^40^ Histological examination of the mouse brain revealed a multifocal ischemic pattern in our model, which corresponds to the widely distributed infarcts observed in human stroke.^41^ We found that infarcts occupied the dorsal-lateral convexity of the parieto-temporal cortex between 0.22 and 2.8 mm posterior to bregma. The mean infarct volume was $1.4\;{\mathrm{mm}}^{3}$ (1.1% of a hemisphere), which was smaller than those of other stroke models. In one study, after permanent coagulation of the distal MCA, the mean infarct volume was $15.4\;{\mathrm{mm}}^{3}$ (12% of a hemisphere).^42^ Moreover, in a study using the fiber model, which is induced by intraluminal monofilament MCA occlusion, the infarct was found to comprise 40%±5% of the hemisphere.^43^

Because of the extensive complexity and variability of the vascular structure of individual mice, matching corresponding vessels and their bifurcations to the literature is difficult. Such variability includes different numbers of MCA branches, variable bifurcation locations, the absence of certain vessel segments, and bifurcation or trifurcation.^44^ These individual differences impact the selection of MCA regions for illumination, which, in turn, affects infarct shapes and distributions.

We performed a 3-D infarct reconstruction to estimate infarct volume and size. The reconstruction revealed spatially separated volumes (Fig. 8), which were presumably supplied by two adjacent MCA branches. Registration of the infarct reconstruction to an MRI-based mouse brain atlas allowed for visualization of its precise anatomical location in relation to other brain structures. This approach may allow for the prediction of potential stroke-associated cognitive and motor deficits, which is especially important for patients.

In summary, we present a new approach that combines OCM and brightfield microscopy for *in vivo* real-time monitoring and analysis of ischemic changes in the mouse brain. Using multimodal stroke region imaging and cranial window implantation, we three-dimensionally visualized blood flow and thrombosis progression during stroke and reperfusion. OCM is a high-resolution imaging technique that allows for microvessel (capillary) circulation monitoring without contrast agents. Extended Bessel beam focusing provides high resolution over a depth of 800  μm, making z axis scanning unnecessary and allowing for high-speed 3-D imaging. We used a modified mouse model of thromboembolic photoinduced stroke, characterized by low mortality and more closely approximating human stroke. This allows for more accurate pharmacological testing. Moreover, such drug treatments could be evaluated with OCM imaging to examine small artery and capillary blood flow dynamics within deeper brain tissues. We demonstrated that, despite superficial artery recanalization, there was a lack of small vessel blood flow in deeper brain regions. The method of 3-D reconstruction and anatomical localization of infarcts provides a new and more precise means of assessing poststroke deficits.

## 5 Appendix

Three supplementary videos are provided. Video S1 is a video showing changes of blood flow during stroke, calculated as total Angio-OCM signal intensity at different depths of the cortex. [URL: https://doi.org/10.1117/1.NPh.7.1.015002.1] Video S2 is a video showing the development of clots and their migration during 20-minute green laser exposure, recorded by a bright field microscope (20×  current speed). [URL: https://doi.org/10.1117/1.NPh.7.1.015002.2] Video S3 is a movie demonstrating infarct regions registered to the high resolution-MRI mouse brain (atlas, frontal sections). [URL: https://doi.org/10.1117/1.NPh.7.1.015002.3]
