# Supplementary material for: In vivo brain imaging with multimodal optical coherence microscopy in a mouse model of thromboembolic photochemical stroke
Source: Neurophotonics. 2020 Jan 22;7(1):015002. doi: 10.1117/1.NPh.7.1.015002 (PMC6977401; doi:10.1117/1.NPh.7.1.015002)
Supplement: Supplementary file 1 [file NPh_007_015002_SD001.pdf]

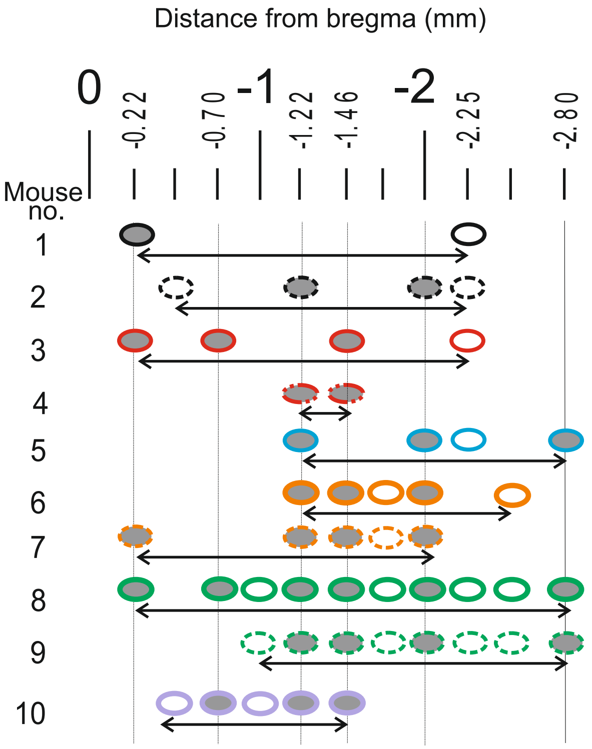

Diagram showing anterior-posterior positions of Fluoro Jade stained infarcts in individual mice. The analyzed subsequent slices (distance between them 200 μm) are shown with each analyzed bregma level indicated on the scale. Circles with gray fulfillment show sections in which the distribution of infarcts (demonstrated in Fig. 7a) was analyzed.
